# Does the 2019 Canada’s Food Guide meet the needs of young athletes?

**DOI:** 10.1177/02601060221093430

**Published:** 2022-04-18

**Authors:** Alexandra J. Heidl, Kathleen Litzenberger, Tamara R. Cohen, Hugues Plourde

**Affiliations:** 1 Faculty of Land and Food Systems, 8166 University of British Columbia, Vancouver, BC, Canada; 2 School of Nutrition, 151165 McGill University, Ste-Anne-de-Bellevue, Quebec, Canada; 3 BC Children’s Hospital Research Institute, BC Children’s Hospital, Vancouver, BC, Canada

**Keywords:** Canada’s Food Guide, young athletes, nutrition, guidelines, energy requirements

## Abstract

**Background:** The Canada’s Food Guide (CFG) encourages Canadians to consume a balanced plate. However, this recommendation may not meet the nutritional needs of young athletes who have increased nutritional requirements. **Aim:** To evaluate how the 2019 CGF can be used to meet the nutritional needs of young athletes. **Method:** Five menu scenarios were created using the CFG’s balanced plate and recipes from Health Canada. Each menu was analyzed to compare nutrient and energy needs of an index athlete (15-year-old male, 71 kg). Estimated energy requirements were based on nutrition guidelines set by National and International sports-nutrition position statements. **Results:** The adjusted CFG balanced plate plus an energy dense beverage at every meal was the closest to meeting the index athlete’s nutrient requirements. **Conclusion:** The 2019 CFG’s balanced plate needs to be adjusted to meet the nutritional requirements of individuals with active lifestyles.

## Introduction

In 2019, Health Canada released a revised version of Canada’s Food Guide (CFG) (Health Canada, 2019a). While the old CFG provided recommendations regarding the number of food servings from four food groups, the revised CFG depicts the average recommended meal as a balanced plate consisting of the following food groups and proportions: 50% fruits/vegetables, 25% whole grains foods, and 25% protein foods. One could argue that the new CFG may be more user-friendly for the average consumer, as it no longer involves keeping track of food group servings. However, from a dietitian’s perspective, the new CFG introduces challenges when having to adjust the balanced plate to the individual needs of the client. Of particular concern are sports dietitians who are not only required to cater nutrition plans to the individual sport, but who must also meet the needs of individual athletes with inconsistent training schedules.

The *Finale des Jeux du Québec* (JQ) (Montreal, QC, Canada) is a multisport event held annually and in alternating summer and winter seasons in Québec (Sports Québec, 2020). The purpose of the JQ is to showcase the talents of, on average, 3330 aspiring young Québec athletes under the age of 18. Throughout the week of competitions, hosts of the event are responsible for providing meals and lodging to participants and facilitators (Sports Québec, 2020). In 2010, it was recommended that SPORT QUÉBEC, an organization responsible for managing the JQ, formulate and implement an evidence-based nutrition policy representative of the original CFG that ensured athletes would be offered nutritious meals for the duration of the competition. Additionally, this policy would include guidelines related to the procurement/selection of external food providers/catering services, with the intention of prioritizing quality of food over cost. The JQ nutrition policy (Drouin-Audet et al., 2013) was introduced in 2011 and revised in 2013 with guidelines based on information presented in both the 2009 Position Statement of Nutrition and Performance released by the Dietitians of Canada and the American College of Sport Medicine (Thomas et al., 2016) and the 2009 Nutrition for Athletes released by the International Olympic Committee (Maughan and Burke, 2012). This policy was then used to create a standardized, user-friendly reference guide intended for use by hosts of the JQ. The release of the 2019 CFG and its new approach to balanced nutrition called for the revision of the JQ nutrition policy; however, it is unknown how this revised CFG would meet the needs of young athletes who participate in the *Finale des Jeux du Québec*. The purpose of this project was to assess how a nutrition policy based on the new CFG could be used to propose a standardized, user-friendly reference guide intended for use by hosts of the JQ. Thus, it was expected that this nutrition policy would support macronutrients and energy needs of adolescents participating in a multiday sporting event.

## Methods

Data from previous work was used to identify the average JQ participant: a 15-year-old male weighing approximately 71kg. Estimated energy requirements and macronutrient distribution for the index participant were calculated using the guidelines published in the 2016 joined position statement by the Academy of Nutrition and Dietetics, Dietitians of Canada (DC), and the American College of Sports Medicine (ACSM) (Thomas et al., 2016) (Table S1) and were aligned with energy estimations of the International Institute of Medicine (IOM) (Thomas et al., 2016). Despite being published in 2016, more recent reviews suggest similar macronutrient ranges (Kerksick et al., 2018). Specifically, following published guidelines available it was determined that the energy requirement or range of macronutrients needed by the average athlete were: 2840-3195kcal/day, 355-710 g carbohydrate (CHO)/day, 85-142 g protein/day and 35-71g fat/day. We suggested that minimum nutrition requirements per meal be set at 830 kcal/meal with a minimum of 20 g of protein/meal. Optional snacks were provided to display an overview of additional calories that would be complementary to increase the athlete’s total energy requirements. Addition of three snacks would increase the total energy to align with the higher requirements of athletes who were participating in more arduous sports. Total energy for three snacks per day to be consumed after meals were set to provide 780 kcal/day, assuming the energy provided by the snacks varies and are not reaching maximum kcal allowed. Specifically, the snacks included a minimum of 50 g CHO and 15 g protein. Individual snacks were set to provide between 260-380 kcal each.

A 5-day sample menu (Scenario I) representative of the new CFG guidelines was created using five randomly selected balanced plate recipes for each meal category (breakfast, lunch, dinner, snack) from the 17 breakfast, 20 snack, and 80 lunch/dinner recipes provided on Health Canada’s website (Health Canada, 2020). Four additional scenarios (Scenario II-V), based on systematic modifications to Scenario I, were developed with the intention of altering nutritional composition to better reflect both recent guidelines and the calculated estimated requirements. All 5 scenarios included the same 3 snacks in their sample meal plans. Each menu item from all 5 scenarios was assessed for caloric, CHO, fat, and protein content according to the Canadian Nutrient File, using the now discontinued software EaTracker^®^ (Dietitians of Canada) to analyze nutrient profiles from portions provided in the recipes. The nutrient analyzes results were then compared to the calculated estimated requirements.

**Plate Scenario Overview:**

Scenario I: Balanced plate (½ fruits/vegetables, ¼ whole grains foods, and ¼ protein foods).
Scenario II: Adjusted balanced plate (¼ fruit, ¼ vegetable, ¼ protein foods, and ¼ whole grain foods).
Scenario III: Adjusted Scenario II - Adjusted balanced plate (¼ fruit, ¼ vegetable, ¼ protein foods, and ¼ whole grain foods) plus an energy dense beverage (i.e. 1 cup 100% fruit juice, 1 cup chocolate milk, 1 cup milk) to consume at every meal.
Scenario IV: “Flipped” balanced plate (½ whole grain foods, ¼ protein foods, and ¼ vegetables and fruit).
Scenario V: Adjusted Scenario IV - “Flipped” balanced plate (½ whole grain foods, ¼ protein foods, and ¼ vegetables and fruit) plus an energy dense beverage (i.e. 1 cup 100% fruit juice, 1 cup chocolate milk, 1 cup milk) to consume at every meal.

## Results

When each scenario was evaluated against the daily recommended nutrient requirements, differences among recommended and calculated energy and macronutrient requirements were revealed. The average energy and macronutrient distribution from each scenario are shown in Table 1. Based on the recommended 2840-3190kcal/ day or 40–45 kcal/kg/day, scenario I balanced plate menu only met 52.6% of the lowest range of the estimated energy requirement. When additional energy dense beverages are added to the menu, the caloric requirements appear to increase and become more aligned with the recommended requirements. This can be seen in scenario III that meets 86.7% of the estimated energy requirements. Similarly, scenario V’s menu aligned with 85.0% of the recommended energy requirements. Despite the addition of energy dense beverages and a “flipped” plate, the requirements are still below the recommendation set by position statements.

**Table 1.** Comparison of 5 Canadian Food Guide (CFG) based scenarios to estimate energy requirements of a 15 year old male athlete weighing 71 kg.

Scenario	Average Energy Distribution Over 5-day Menu			
	Calories (kcal/kg/d)	Protein (g/kg/d)	Carbohydrate (g/kg/d)	Fat (%)
I: Balanced plate = CFG + 3 snacks / day	21.03	1.22	2.95	29.2
II: Adjusted balanced plate = 1/4 portion of each food group	31.23	1.68	4.69	23.8
III: Scenario II + 2 cups juice + >20g protein	34.69	1.73	5.42	21.4
IV: “Flipped” balanced plate = ½ whole grains	30.68	1.71	4.47	23.9
V: Scenario IV + 2 cups juice + >20g protein	34.12	1.77	5.45	21.5
Estimated Nutritients Requirements	40-45	1.2-1.8	6-10	20-35

For macronutrients, the menus from scenarios I and IV were sufficient in meeting the minimum protein requirements of 85g/ day, but not all the meals provided >20 g protein indicating an uneven distribution of proteins through the menus. As for fat, scenario IV and V provided insufficient energy from fat when compared to recommendations. Finally, the balanced plate model was found to meet 58.2% of the estimated minimum requirements for CHO, whereas the “flipped” plate (scenario IV) met 72.8% of CHO needs compared to recommendations.

Each scenario included 3 snacks/day that were identical to those provided in scenario I. However, we found that the snacks did not meet the recommended 20 g of protein, nor the 50 g of CHO.

## Discussion

This project suggested that implementing the balanced plate and the associated recommendations of the new CFG into the *Finale des Jeux du Québec* food policy did not provide adequate CHO, fat and energy for average athletes participating in these events. This is demonstrated in the Scenario I menu that reflects the balanced plate model, where only half of the estimated energy and CHO requirements for the average participants were met. Additionally, the recommended 3 snacks per day provided on average 480 kcal, which is below the recommended 780 kcal set by various position statements (Maughan and Burke, 2012; Maughan and Burke, 2016; Thomas et al., 2016). This project showed that adjusting the new CFG helped increase the nutrient content of the meals, thus providing adequate energy for the sample athlete. As mentioned, the adjusted menu Scenario III was most closely aligned with the new CFG and the energy requirements for the index athlete. However, despite this being the menu “closest” to the new CFG recommendations, this menu also did not meet the energy or CHO requirements. Further, the new CFG suggests limiting intake of sugary drinks, such as fruit cocktails and sports drinks that contribute to excess free sugar (Health Canada, 2019b). However, there are no recommendations suggesting that 100% fruit juices containing natural fruit sugars could not be considered in supporting the energy needs of young athletes. This project explored the concept of an “adjusted-flipped CFG”, as seen in Scenario V (plate proportions at each meal: ½ whole grain foods, ¼ protein foods, ¼ vegetables and fruit, plus an energy dense beverage). This scenario was the second menu that came closest to providing adequate nutrients. Although Scenario V was comparable to scenario III in terms of nutrient content, the “flipped” plate, with an increase of whole grain foods and decrease in vegetables, does not reflect the new CFG. It is important that dietitians who work in creating nutrition policies or guidelines, that pertain to athletes, consider adjusting their recommendations as we are suggesting here (i.e. Scenario III of the balanced plate and provide additional food items that would help increase the caloric content). As policies such as the JQ are evidence based and reflect both Health Canada and the organization’s goals, we suggest that policy makers and dietitians consider the macronutrient and energy needs when working on menu planning for intended young athletes. Additionally, based on the organization and their educational goals, other considerations may include: 1) offering additional food items such as 100% fruit juice or whole fruit 2) offer no more than 2 servings of lean red meat per week, which includes avoiding processed meats such as cold cuts.

As the CFG is a tool meant to be used by the general population, including educators, health care providers and the public, it is essential that the guidelines and messages it conveys be easy to follow. It is also important that the dietary recommendations it conveys meet the energy requirements of the average Canadian who partakes in different activity levels, and not just sedentary individuals. This project highlighted a limitation of the new CFG based on its utilization to update the nutrition policy for the *Finale des Jeux du Québec*. These limitations include the difficulties in quantifying portion sizes for varying age groups and activity levels, and how to use the guide when mixed dishes are included in meals. Further, it was not clear how 100% fruit juice (no sugar-added) should be implemented in meal planning for highly active individuals.

This project is limited by way that we only examined the nutrition needs of young athletes and used the information available from the official agency website. Conversely, it raises a point of concern that there needs to be adjustments made within the CFG for all Canadians who engage in >150 min of moderate-to intensive physical activity per week. Findings from this project can be used by other sport organizations that also use CFG to create their own nutrition policies when organizing sport tournaments and educating the sport community on the nutritional needs of athletes. While the revised CFG is easy to understand, it is important that dietitians consider their population needs as “one size does not fit all”.

## Supplemental Material

sj-docx-1-nah-10.1177_02601060221093430 - Supplemental material for Does the 2019 Canada’s Food Guide meet the needs of young athletes? Click here for additional data file. Supplemental material, sj-docx-1-nah-10.1177_02601060221093430 for Does the 2019 Canada’s Food Guide meet the needs of young athletes? by Alexandra J. Heidl, Kathleen Litzenberger, Tamara R. Cohen and Hugues Plourde in Nutrition and Health
