# Supplementary material for: Does the 2019 Canada’s Food Guide meet the needs of young athletes?
Source: Nutr Health. 2022 Apr 18;28(3):297–300. doi: 10.1177/02601060221093430 (PMC9379377; doi:10.1177/02601060221093430)
Supplement: sj-docx-1-nah-10.1177_02601060221093430 - Supplemental material for Does the 2019 Canada’s Food Guide meet the needs of young athletes? [file sj-docx-1-nah-10.1177_02601060221093430.docx]

**TABLE 2: Comparison of nutrient recommendations for young athletes based on sports position statements and dietary guidelines published from 2009 to present (2020)**

|  | **Joint Position Statement**  **(ACSM, ADA, DC)^4^** | **International Olympic Committee, (IOC)^5^** | **IOC Nutrition for Athletes^8^** | **Sports Nutrition Needs for Child and Adolescent Athletes^9^** | **International Society of Sports Nutrition (ISSN)^10^** | **Nutrition for Special Population: Young, Female, and Masters Athletes^11^** |
| --- | --- | --- | --- | --- | --- | --- |
| ***Year*** | ***2016*** | ***2009*** | ***2016*** | ***2016*** | ***2018*** | ***2019*** |
| **Energy** | Based predictive equations, individual |  |  | 14-18y active  F: 2400kcal/d  M: 2800-3200kcal/d | 40-70kcal/kg/d for athletes 50-100kg (mod intensity:  2-3h/d 5-6x/wk) |  |
| **CHO** | 6-10g/kg/d |  | 6-10g/kg/d for endurance program (1-3h/d of moderate to-high intensity exercise) | 12-18y athletes  F: 3-5.5g/kg/d  M: 6-9g/kg/d | 5-10g/kg/d  (400-1500g/d for  50-150kg athlete) |  |
| **Protein** | 1.2-1.7g/kg/d | 1.2-1.6g/kg BW/d  (min 0.8g/kg/d,  max 1.7g/kg/d) | 1.2-1.8g/kg BW/d | 3-18y athletes  1.2-1.8g/kg/d | 1.2-2.0g/kg/d  (60-300g/d for 50-150kg athlete) | 1.5g/kg/d |
| **Fat** | 20-35% of total energy intake |  |  | 20-35% | 0.5-1g/kg/d |  |
| **Fluids** | 450–675ml/lb weight loss | Rehydration: 1.2-1.5 L fluid/kg weight loss |  | Replacement during exercise (mL/hr):  Kg BW x 13  Replacement after exercise (mL/hour):  Kg BW x 4 |  |  |
| **Pre-effort snack** | Lots of fluids  Low in fat & fiber  High in carb  Mod. protein | 1-5g/kg BW 0-6h pre-competition | 50-75g CHO + 5-20g Protein  OR 1-4g/kg BW |  |  |  |
| **During**  **effort** | Replace fluid losses  30-60g CHO/h if effort >90min |  | 30-60g/h of CHO for endurance and “stop and start” sports 1-2.5h |  | 0.7g/kg/h |  |
| **Post- effort snack** | 1.0-1.5g/kg in first 30min and  q 2h 4-6h post effort | 1g/kg BW/h 0-4 h post competition | 1g/kg BW 0-4h  for rapid refueling post effort |  | 1.2g CHO/kg/h  0-4h in recovery | 0.11g/kg/h in recovery |

**Abbreviations**: ACSM: American College of Sports Medicine, ADA: American Dietetic Association, CHO: Carbohydrate, DC: Dietitians of Canada, IOC: International Olympic Committee, ISSN: International Society of Sports Nutrition

**References:** ^4^ Thomas, Erdman, and Burke L (2016). ^5^Maughan and Burke (2012). ^8^ Maughan R and Burke L (2016). ^9^ Kerksick, Fox, and Fox E (2016). ^10^ Kerksick et al., (2018). ^11^ Desbrow et al. (2019).
